# Response of rumen fermentation and microbiota to dietary supplementation of sodium selenite and bio-nanostructured selenium in lactating Barki sheep

**DOI:** 10.1186/s12917-023-03799-7

**Published:** 2023-11-27

**Authors:** Alaa Emara Rabee, Mayada M. H. Khalil, Galal Abou Khadiga, Ahmed Elmahdy, Ebrahim A. Sabra, Mohsen A. Zommara, Ibrahim M. Khattab

**Affiliations:** 1 Animal and Poultry Nutrition Department, Desert Research Center, Cairo, Egypt; 2 Department of Animal and Fish Production, Faculty of Desert and Environmental Agriculture, Matrouh University, Matrouh, Egypt; 3 Departement of Poultry Production, Faculty of Desert and Environmental Agriculture, Matrouh University, Matrouh, Egypt; 4 Department of Dairy Science, Faculty of Desert and Environmental Agriculture, Matrouh University, Matrouh, Egypt; 5 Animal Biotechnology Department, Genetic Engineering and Biotechnology Research Institute, University of Sadat City, El-Sadat City, Egypt; 6 Dairy Science Department, Faculty of Agriculture, Kafrelsheikh University, Kafr El- Sheikh, Egypt

**Keywords:** Sheep, Green chemistry, Organic selenium, Rumen, Bacteria, Archaea

## Abstract

Dietary selenium (Se) sources affects the structure of the rumen microbial community and rumen fermentation. This study evaluated the effects of sodium selenite (SS) and bio-nanostructured selenium (SeNSM) on rumen fermentation and structure of rumen microbial community of lactating Barki ewes. Twenty one lactating Barki ewes were assigned into three groups based on their body weight and milk yield. The experiment lasted for 50 days, whenever, the control group was fed basal diet; group SS received basal diets plus sodium selenite as inorganic source of Se; and group SeNSM received basal diet plus organic selenium bio-nanostructured. Ruminal pH and volatile Fatty Acids (VFA) was lower (*P* < 0.05) in SeNSM group compared to control. Principle Coordinate Analysis separated the microbial communities into three clusters based on feeding treatment. The bacterial community was dominated by phylum Bacteroidetes and Firmicutes that were affected (*P* < 0.05) by Se sources. Specifically Bacteriodetes was higher (P < 0.05) in SS and SeNSM groups; and Firmicutes was higher (P < 0.05) in the control group. Moreover, the predominant bacterial genera were *Prevotella*, *Rikenellaceae RC9 gut group*, *Unclassified_Bacteroidales,* which were higher (*P* < 0.05) in SeNSM group. The methanogenic community belonged to phylum Euryarchaeota and was significantly decreased (P < 0.05) by Se supplementation. Principal component analysis based on rumen fermentation parameters, and relative abundances of bacteria and methanogens revealed three distinct clusters. These findings suggest that Se supplementation affected the relative abundances of dominant bacterial groups, declined rumen methanogens and SeNSM supplementation showed some positive impacts on some fibrolytic bacteria.

## Introduction

Balanced diets are essential for the health and performance of animals. The diet of lactating animals should contain proper amounts of macro and micro minerals that are required for maintaining health and optimum milk production. The diets of ruminant animals are originated mainly from forage plants, which vary in their contents of minerals. Moreover, the mineral content in the plant depends on several factors such as soil, plant type, climate conditions, and agricultural practices [1]. Subsequently, the dietary content of trace minerals, including selenium (Se), can be varied and animal diet should be supplemented by trace minerals to maintain animal optimum health and production performance [2, 3].

Selenium plays a vital role in animal productivity as it is involved in several metabolic functions and represents the metal part of several enzymes [4]. Selenium also has antioxidant properties and can boost the immune system [5]. Thus, the deficiency of Se declines productivity and causes weakness in offspring besides several reproductive disorders in males and females [6]. Therefore, the animal diet should maintain a suitable level of bioavailable Se [7]. Selenium could be supplemented in organic or inorganic forms. According to Ahuja and Parmar [8], the normal dietary requirement of Se is approximately 0.1–0.3 ppm of DM intake in ruminants. Inorganic Se, such as sodium selenite, has a lower bioavaliblilty than organic forms such as lactate-protein Se complex, Se-proteinate, Se-enriched yeast, and Se-enriched alga [9]. Moreover, organic Se is distinguished from inorganic Se by its higher-safety level [7–10].

Furthermore, nanotechnology was used to produce bio-Se nanoparticles (SeNPs) to improve the bioavailability of Se [11]. Organic nano Se was produced using several microorganisms like *Streptococcus thermophilu* and *Lactobacillus delbrueckii subsp. Bulgaricus,* and enhanced the immunity and performance of pregnant ewes and growing lambs [3–13]. Hendawy et al. [14] highlighted the effectiveness of SeNPs and recommended further research into their effects on the rumen microbial community. Therefore, It is necessary to understand deeply the effect of different sources of Se on animal performance, health, and rumen fermentation.

Bacteria make the greatest contribution to rumen fermentation [15]**,** which is affected mainly by the animal diet [16]**.** Consequently, it is necessary to study the effect of feeding interventions on rumen microbial fermentation. Minerals affect the rumen ecosystem by changing the rumen dilution rate and osmotic pressure [17–19]**.** Previous studies [20, 21] reported that Se supplementation improved rumen fermentation, volatile fatty acid (VFA) production, and microbial population.

Using organic Se in the form of Se-Yeast increased short-chain fatty acids in the rumen compared to inorganic Se [19]. In addition, inorganic Se has lower bioavailability for rumen microorganisms [21–23]**.** However, limited research has examined the effect of Se sources on the modulation and structure of rumen microbial groups. Tian et al. [17] and Cui et al. [18] observed that organic Se improved the antioxidant activity in the rumen, affected rumen bacteria and protozoa, and improved rumen fermentation. Cui et al. [18] investigated the effect of different levels of Se on rumen fermentation and microbial communities in Tibetan sheep and found that increasing the level of organic Se was associated with improvement in rumen fermentation and increament in the relative abundances of major bacterial groups such as Bacteroidetes and Firmicutes. The FDA set the selenium supplementation rate at 0.3 mg Se/kg in diet for sheep. In countries such as Egypt, where the soil suffers from a lack of Se [24], and in cases where specific energy requirements increase (e.g., early lactation), it requires increasing the dose of Se [3]. Recent studies have reported that super nutritional organic Se supplementation (> 1 mg/kg DM) found to enhance production performance and antioxidant status in sheep and goats [3, 21, 25]. However, the role of organic and inorganic Se in modulating the diversity and composition of rumen bacteria is still poorly understood. Therefore, this study aims to get insights into the effect of inorganic and bio-Se nanoparticles on rumen fermentation and changes of rumen microbiota in Barki ewes.

## Materials and methods

### Study location

This experiment was conducted at the farm of the Faculty of Desert and Environmental Agriculture, Matrouh University, Matrouh, Egypt.

### Bio-nanostructured selenium preparation

Bio-nanostructured selenium (SeNSM) were prepared according to the method of Prokisch and Zommara [26] and as described in our previous study [3]. Briefly, *Lactobacillus delbrueckii subsp. bulgaricus* and *Streptococcus thermophilus* were mixed in yogurt culture at 1:1 (w/w) and cultivated in whey yeast extract media [27]. Afterwards, 100 ppm of Se as sodium selenite (Sigma-Aldrich, Switzerland) was added to the media and incubated (72 h, 37 °C) the culture media were centrifuged (4500 g, 20 min, 10 °C). Tris-HCl buffer was used to wash the sediment two times, then with distilled water, as described by Prokisch, et al. [28]. The sediment was air dried (60 °C, 24 h) and then milled to a fine powder to obtain the purified SeNSM product. A gram of SeNSM final dry product contains 7.5 mg Se and 55 to 238 nm as particle size.

### Animal management and experimental design

This study is a apart of our previous study [3] and animal management and feeding for the present study have been reported previously. In Brief, 21 Barki ewes (*Ovis aries*; average body weight, 32.72 ± 0.84 kg; 14 ± 7 days in lambing date) were used in this study for 50 days as an experimental period. Animals were divided into three groups according to their BW and milk yield from a previous lactation period to receive one of three experimental diets: basal diet (Control); basal diet supplemented with 1.2 mg/kg dry matter (DM) of Se from sodium selenite (analytical grade) as the inorganic source of Se (SS); basal diet supplemented with 1.2 mg/ kg DM of Se from SeNSM as the organic source of Se (SeNSM). The Se supplementation was mixed daily into the concentrate before feeding and then mixed with roughage. According to our previous study [3] ewes in the control, SS, and SeNSM groups received 1.11, 2.32, and 2.35 mg of Se /h /d, respectively. The animals were housed individually in shaded pens (1 m × 1.5 m) with free access to water. Diets were offered a fixed amount as % BW according to NRC [29] to each ewe individually two times a day at 08.00 and 17.00 h. The ingredients and chemical composition of the basal diet are shown in Table 1.

**Table 1:** Ingredients and nutritional composition of the basal diet

Item	Contents
Ingredients, g / kg DM	
Berseem hay	500
Yellow corn	262
Soybean meal	65
Wheat bran	160
Limestone	10
Vitamins and minerals ^a^	3
Nutrient composition	
Dry matter, g/kg as-fed	894
Crude protein, g/kg DM	157
Neutral detergent fiber, g/kg DM	424
Acid detergent fiber, g/kg DM	229
Net energy of lactation^b^, Mcal/kg	1.66
Selenium, mg/kg	0.06

^a^ Composition: Each 1 kg consists of 10,000,000 IU vitamin A, 2000000 IU vitamin D, 10000 IU vitamin E, 45.8 g di calcium phosphate, 15 g magnesium sulfate, 6.15 g ferrous sulfate, 0.393 g potassium iodide, 0.753 g copper sulfate, 0.248 g cobalt sulfate, and 0.373 g zinc sulfate

^b^ Estimated using the NRC (2007)

Diet was analyzed for for DM (method 930.15) and nitrogen (method 954.01) according to the procedure of to the Association of Official Analytical Chemists [30]. Neutral detergent fiber (NDF) and acid detergent fiber (ADF) were determined as described by Van Soest et al. [31]. Analyses of NDF were performed using α-amylase and expressed exclusive of residual ash using an ANKOM 200 Fiber Analyser unit (ANKOM Technology Corporation, Macedon, USA). The ADF concentrations were expressed exclusively of residual ash. Concentrations of Se in diet samples were measured using graphite furnace atomic absorption spectrometry, following the method described by Elmer [32].

### Sampling and analysis of rumen fluid

By the end of the experimental period, ruminal liquor samples were withdrawn from all ewes before the morning feeding using a stomach tube with GM-0.336A vacuum good pump (Jinteng, Tianjing, China). Ewes were restrained without anesthesia or sedation and released. The first 50 mL of rumen fluid was discarded to minimize saliva contamination according to El-Essawy et al. [33]. Ruminal fluid was collected after filtering through 4 layers of cheesecloth and 10 ml aliquot of ruminal fluid was immediately used to extract DNA. pH was measured directly using a pH meter (Accumet Model 15, Fisher Scientific, USA). Another 40 ml of the ruminal fluid was acidified with 2.5 mL of 6 N HCl and frozen (− 20 °C) for further analysis of ruminal ammonia and total VFA. Ammonia-N concentrations were measured colorimetrically by spectrophotometer (Alpha-1101 model; Labnics Equipment, California, USA) using commercial lab test (SPINREACT, Ctra. Santa Coloma, 7, Girona, Spain). Total VFA concentrations were determined according to the procedure of Warner [34].

### Analyses of microbial communities

DNA was extracted from one milliliter of rumen sample. The sample was centrifuged at 13,000 rpm, and the remained pellets were used in DNA extraction by i-genomic Stool DNA Extraction Mini Kit (iNtRON Biotechnology, Inc.) according to the manufacturer’s instructions. The quality and quantity of extracted DNA were verified by Nanodrop spectrophotometer and gel electrophoresis. The composition and diversity of microbial community were studied using amplification of variable V4 region on 16S rDNA gene by 515F and 926R primer sets using the following PCR conditions: 94 °C for 3 min; 35 cycles of 94 °C for 45 s, 50 °C for 60 s, and 72 °C for 90 s; and 72 °C for 10 min. The purified PCR-amplicons were sequenced using Illumina MiSeq system at Integrated Microbiome Resource (IMR, Dalhousie University, Halifax, NS, Canada).

### Bioinformatics and statistical analyses

The generated paired-end (PE) Illumina raw sequences were analyzed in R (version 4.2.2) using the DADA2 pipeline [35]. The Fastq files of Paired-end reads were demultiplexed and their quality was checked. Then, the sequences were filtered, trimmed, and dereplicated followed by merging read 1 (R1) and read 2 (R2) reads together to get denoised sequences. The denoised sequences were subjected to removing the chimeras; then Amplicon Sequence Variants (ASVs) were generated. Taxonomic assignment of ASVs was conducted using “*assign Taxonomy*” and “*addSpecies”* functions and microbial taxa were identified using SILVA reference database (version 138). Alpha diversity indices, Chao1, Shannon, and InvSimpson, were calculated. Moreover, Beta diversity of microbial communities was dtermined as principal coordinate analysis (PCoA) using bray–curtis dissimilarity. The differences in rumen fermentation parameters, microbial diversity indices, and the relative abundances of bacterial phyla and genera were determined by One-way Anova using Duncan test a *P* < 0.05 as a threshold of statistical significance. Morover, Principal component analysis (PCA) was determined using the relative abundance of dominant bacterial phyla and genera and rumen fermentation to compare the clustering of individuals of every sheep group.

## Results

### Rumen fermentation

Ruminal pH, ammonia-N, and total VFA, are shown in Table 2. Ruminal pH was higher in control group; while VFA was higher (*P* < 0.05) in SeNSM group compared to control.

**Table 2:** Effect of selenium sources on rumen fermentation paramters of lactating Barki ewes

Item	Treatments^1^			SEM	*P*-value
	Control	SS	SeNSM		
pH	6.72^a^	6.57^ab^	6.47^b^	0.04	0.04
Ammonia-N, mg/dl	12.71	11.25	10.91	0.54	0.39
Total VFA, mM	81.83^b^	84.11^ab^	85.50^a^	1.35	0.03

^1^ Control = diet without supplementations; SS = diet supplemented with SS at 1.2 mg/kg DM, SeNSM = diet supplemented with SeNSM at 1.2 mg/kg DM

^a,b^ Means within a row with different subscripts differ significantly (*P* < 0.05)

SEM = standard error of the mean

### The diversity of rumen microbial communities

**Fig. 1:**
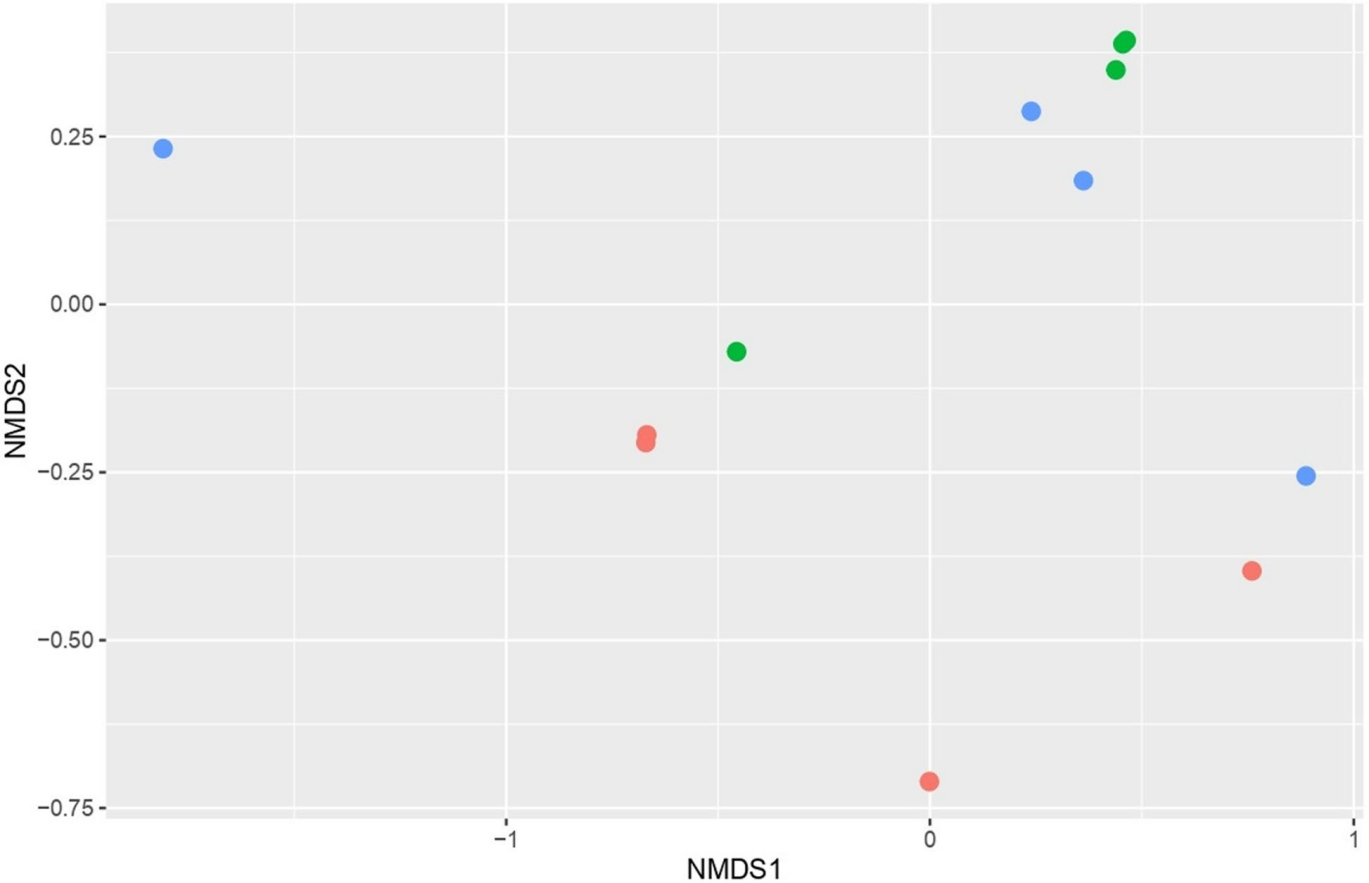
Principal coordinates analysis (PCoA) of microbial communities in the rumen of lactating Barki sheep as affected by dietary selenium sources based on Bray–Curtis dissimilarity. The PCoA was conducted between three sheep groups, red circles for control group, green circles for SS group, and blue circles for SeNSM group

The Illumine sequencing of the V4 region on the 16S rDNA gene in all rumen samples generated 393,443 high-quality sequence reads with a mean of 32,786.9 ± 2257.93 (Mean ± standard error (SE)). The total sequence reads in control group was 129,653 with an average of 32,413.25 ± 4735.69, the total sequence reads in SS group was 124,464 with an average of 31,116 ± 4527.28, and the total sequence number in SeNSM group was 139,326 with an average of 34,831.5 ± 3284.4. Different alpha diversity indices were measured; the number of ASVs and Chao1 index were higher in SeNSM group compared to other groups; also Shannon and InvSimpson indices were higher in SS group compared to other groups (Table 3). The beta diversity of microbial communities in the rumen of sheep groups was calculated using Principle Coordinate Analysis (PCoA) based on Bray Curtis dissimilarity metrics (Fig. 1). The results revealed that the selenium source separated the microbial communities based on animal diets.

**Table 3:** Effect of selenium sources on the microbial alpha diversity indices in the rumen of lactating Barki sheep

	Treatments^a^			SEM	*P*-value
	Control	SS	SeNSM		
	Mean	Mean	Mean		
Observed ASVs	341.25	385.75	388.5	18.41	0.54
Shannon	4.32	4.48	4.32	0.11	0.82
Chao1	343.15	391.25	394.25	19.47	0.53
Invsimpson	30.48	36.51	32.20	4.18	0.85

^a^Control = diet without supplementations; SS = diet supplemented with SS at 1.2 mg/kg DM, SeNSM = diet supplemented with SeNSM at 1.2 mg/kg DM. ASV = amplicon sequence variants. SEM = standard error of the mean

### Structure of microbial communities

The sequencing failed or was weak in six samples, and counterpart samples were discarded to keep an equal sample size (4 samples per group). Inspection of the bacterial community revealed 12 bacterial phyla and one archaeal phylum (Table 4). Moreover, 0.07% of sequence reads was not classified. Phylum Firmicutes and Bacteroidetes dominated the bacterial community and represented together 88% of sequence reads. Other bacterial phyla that made up more than 1% of the bacterial community were Planctomycetota and Proteobacteria. Additionally, bacterial phyla that represented less than 1% were Cyanobacteria, Desulfobacterota, Verrucomicrobiota, Actinobacteriota, Armatimonadota, Chloroflexi, and Spirochaetota. Phylum Fusobacteriota was found only in SeNSM group, and phylum Synergistota was not detected in control group (Table 4). Selenium source affected the relative abundance of dominant bacterial phyla significantly (*P* < 0.05), wherever phylum Bacteroidetes was increased significantly and SS group showed the highest relative abundance of this phylum followed by SeNSM and control group, respectively (Table 4).

**Table 4:** Effect of selenium sources on the relative abunadance (%) of bacterial phyla in the rumen of lactating Barki sheep

	Treatments^1^			SEM	*P*-value
	Control	SS	SeNSM		
	Mean	Mean	Mean		
Firmicutes	59.6^a^	27.31^b^	26.99^b^	6.28	0.021
Bacteroidetes	23.3^b^	69.6^a^	61.7^a^	7.53	0.003
Planctomycetota	7.37	0.53	4.88	1.42	0.10
Proteobacteria	4.70	0.18	3.25	1.42	0.42
Cyanobacteria	0.63	1.04	0.53	0.12	0.24
Fusobacteriota	ND	ND	0.6	ND	ND
Desulfobacterota	0.16	0.19	0.31	0.041	0.37
Verrucomicrobiota	0.35	0.35	0.51	0.042	0.25
Actinobacteriota	0.60	0.042	0.71	0.16	0.19
Armatimonadota	0.08	0.05	0.04	0.009	0.14
Chloroflexi	0.38	0.18	0.19	0.052	0.20
Spirochaetota	0.04^b^	0.23^a^	0.09^b^	0.034	0.0001
Synergistota	ND	0.11	0.003	ND	ND
Euryarchaeota	3.47^a^	0.31^b^	0.13^b^	0.56	0.006

^1^ Control = diet without supplementations; SS = diet supplemented with SS at 1.2 mg/kg DM, SeNSM = diet supplemented with SeNSM at 1.2 mg/kg DM

^a,b^ Means within a row with different subscripts differ significantly (*P* < 0.05)

SEM = standard error of the mean; ND = not detected

Phylum Bacteroidetes was dominated by family Prevotellaceae, Unclassified_Bacteroidales, and Rikenellaceae. Family Prevotellaceae and Rikenellaceae were predominated by genus *Prevotella* and *Rikenellaceae RC9* gut group, respectively (Table 5). These genera besides Unclassified_Bacteroidales, showed their highest relative abundance in sheep SeNSM group. Phylum Firmicutes decreased significantly (*P* < 0.05) in the rumen of supplemented groups (SS, and SeNSM) compared with the control group (Table 4). On the family level, the majority of phylum Firmicuteds was classified into Oscillospiraceae, Christensenellaceae, Lachnospiraceae, Ruminococcaceae, Peptostreptococcaceae, and Peptostreptococcaceae. On the genuse level, phylum Firmicuteds was dominated by *Christensenellaceae R-7 group*, *Unclassified_Lachnospiraceae*, and *Oscillospiraceae NK4A214 group* that were decreased significantly by selenium supplementation (*P* < 0.05) (Table 5).

**Table 5:** Effect of selenium sources on the relative abunadance (%) of dominant bacterial genera in the rumen of lactating Barki sheep

	Treatments^a^			SEM	*P*-value
	Control	SS	SeNSM		
	Mean	Mean	Mean		
**Phylum: Firmicutes**					
Oscillospiraceae NK4A214 group	15.84^a^	8.79^c^	11.54^b^	0.68	0.01
Christensenellaceae R-7 group	13.09^a^	7.71^b^	6.54^b^	0.82	0.01
Unclassified_Lachnospiraceae	3.24	3.77	2.64	0.42	0.58
Oscillospiraceae UCG-002	0.77	1.58	0.87	0.18	0.12
Lachnospiraceae XPB1014 group	0.96	0.83	0.59	0.14	0.64
Unclassified_Ruminococcaceae	1.57	0.56	1.22	0.22	0.20
Lachnospiraceae NK3A20 group	1.23	0.65	0.42	0.22	0.39
Unclassified_Christensenellaceae	0.44	0.59	0.51	0.09	0.82
Butyrivibrio	0.56	0.39	0.32	0.09	0.62
**Phylum: Bacteroidia**					
Unclassified_Bacteroidales	3.91	10.86	13.00	2.48	0.36
Prevotella	16.72^b^	35.12^a^	37.5^a^	2.181	0.001
Rikenellaceae RC9 gut group	6.75^b^	7.23^b^	14.48^a^	0.87	0.01
Prevotellaceae UCG-003	0.72^c^	2.41^a^	1.73^b^	0.26	0.02
**Phylum: Planctomycetota**					
Unclassified Pirellulaceae	3.55	0.89	1.99	0.85	0.52
p-1088-a5 gut group	0.99	0.17	0.58	0.15	0.09
**Phylum: Euryarchaeota**					
Methanobrevibacter	3.42^a^	0.29^b^	0.13^b^	1.30	0.006
Methanosphaera	0.05	0.013	ND	ND	ND

^a^Control = diet without supplementations; SS = diet supplemented with SS at 1.2 mg/kg DM, SeNSM = diet supplemented with SeNSM at 1.2 mg/kg DM

^a,b,c^ Means within a row with different subscripts differ significantly (*P* < 0.05)

SEM = standard error of the mean; ND = not detected

**Fig. 2:**
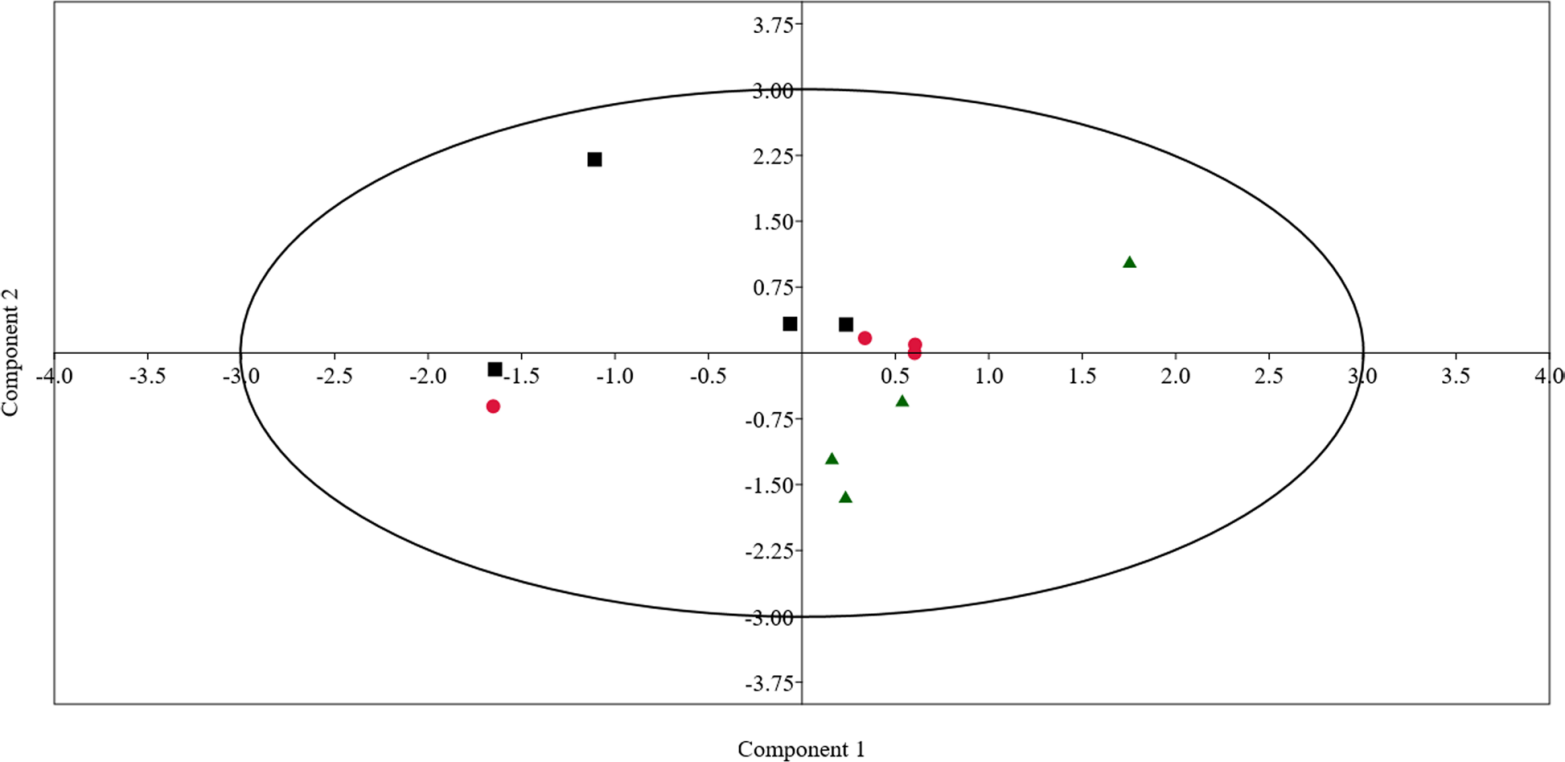
Principal component analysis (PCA) based on rumen fermentation parameters and the relative abundances of rumen bacterial phyla, and dominant ruminal bacterial genera of three sheep groups fed different diets; black squares for ontrol group, red circles for SS group, and black triangles for SeNSM group

Several bacterial genera were detected exclusively in a specific group such as *Butyrivibrio fibrisolvens, Alloprevotella, Bacteroides;* that were not detected in control group. Moreover, g*enus Clostridium* sensu stricto *1, Cellulosilyticumthat, Staphylococcus Brevundimonasthat* were not detected in SS and SeNSM. Phylum Spirochaetota was significantly higher (*P* < 0.05) in SS group compared to Control and SeNSM. Phylum Synergistota was found only in sheep SS group. Furthermore, the methanogenic community was assigned to phylum Euryarchaeota, which was represented 1.3% of total sequence reads and was decreased significantly (P < 0.05) by Se supplementation. Euryarchaeota was assigned to genus *Methanobrevibacter* and genus *Methanosphaera* (Table 5), *Methanobrevibacter* was decreased significantly (P < 0.05) due to Se supplementation. *Methanosphaera* was not detected in the SeNSM group. Principal component analysis (PCA) (Fig. 2) was conducted based on the relative abundance of dominant bacterial phyla, genera and rumen fermentation parameters. The result of PCA revealed that animals of control group were separated from supplemented groups (SS and SeNSM) and the differences between animals were driven by the relative abundances of phylum Bacteroidetes and Firmicutes, and genus *Oscillospiraceae NK4A214 group* and *Prevotella*.

## Discussion

In our previous study, SeNSM was produced by yogurt culture containing *Streptococcus thermophilu* and *Lactobacillus delbrueckii* subsp. bulgaricus in a whey yeast extract media [3]. Furthemore, SeNSM can be used as organic nano- Se supplementation to improve animal health and productivity. The bioavailability of Se, affects rumen fermentation, animal health, and productivity. Therefore, it is necessary to examin the emerging sources of Se such as organic nano Se that showed higher bioavialbility and safty compared with inorganic Se [36]. Organic and nonorganic forms of Se were studied in the context of their effect on rumen fermentation, and animal health and productivity [18–24, 26–36]. However, their effect on diversity and structure of microbial communities has received less attention [14–17]. Previous studies on lactating goats and Holstein cows [37, 38] a long with our previous study on lactating ewes [3] indicated that no effect of nano Se on DM intake. However, there is a significant improvement in milk production, lamb performance and nutrient digestibility, which indicated to an improvement in rumen fermentation [3]. Numerically, adding Se increased total VFA concentration and decreased ruminal pH, while organic Se had a significant effect on increasing total VFA concentration and decreased ruminal pH compared to the control in the current study. In agreement with these results, previously published studies support that organic Se supplementation increased total VFA concentration [18–22, 37, 38]. The improvement in VFA production due to Se supplementation could be attributed to the increase in the digestion of dietary complex carbohydrates [3] due to higher bioavailability of SeNSM and a greater improvement in the antioxidant status of the rumen promote more nutrients to be fermented by the cellulolytic bacteria [18]. Also, propionic acid production in the rumen suppresses methane production, improving animals’ feed efficiency [19]. Moreover, Taheri et al. [39] explained that Se supplementation improves the energy metabolism in the body.

Diet composition is the main determiner of the diversity and composition of the rumen microbial community. The results of PCoA (Fig. 1) showed that Se sources separated rumen microbial communities in the rumen of lactating sheep. However, the differences in alpha diversity indices (Table 3) were not significant, which agrees with a study on sheep supplemented with organic Se [18]. Rabee et al. [15] reported changes in microbial diversity in the rumen of Barki due to changing the diet**.** Previous studies on sheep and goat [17, 18] showed that the rumen bacterial community is dominated by phylum Bacteroidetes and Firmicutes, which support our results. Supplementation with SS and SeNSM increased the relative abundance of the phylum Bacteroidetes, the most predominant phylum, compared to the control group. The improvement was particularly evident in the SS group. Kim et al. [40] reported that Se promotes bacterial reproduction, which might explain our finding.

Previous studies [15–24, 26–38, 40, 41] indicated that members of Bacteroidetes are responsible for the utilization of different substrates such as protein and polysaccharides, including cellulose, and hemicellulose. Consequently, it could be expected that Se supplementation, could induce the digestion of complex polysaccharides in the rumen [18]. This speculation is supported by the higher relative abundance of genus *Prevotella*, *Rikenellaceae RC9 gut group*, and Unclassified_Bacteroidales in SeNSM group, which is in the line with a previous study on goats [17]. Genus *Prevotella* dominated the microbial communities of several ruminant species and it plays an active role in the utilization of amino acids and hemicelluloses, and pectin and produces propionate and xylanase [42, 43]. Moreover, genus *Rikenellaceae RC9 gut group*, which showed higher relative abundance in SeNSM group, plays a critical role in the digestion of crude fiber [44, 45]. Furthermore, phylum Spirochaetes, that was higher in SS group, has a potential role in fiber digestion in the rumen, which highlight Se supplementation in enhancing different fibrolytic microbial groups [46].

These findings are supported by the results of Tian et al. [17] who showed that Yeast-Se improved the expression of carbohydrate metabolism pathways in the rumen of goats. On the other side, Aguilar-Marin et al. [47] linked the lower methane emission with the higher relative abundance of genus *Prevotella* in the rumen of buffalo. Genus *Prevotella* utilizes hydrogen, the primary substrate for methanogenesis, to produce propionic acid, which affects methane production adversely [15–24, 26–38, 40–48].

In this context, the relative abundance of rumen methanogens, phylum Euryarchaeota, was declined in supplemented sheep groups SS and SeNSM, whenever, the relative abundance of *Prevotella* was increased, which agrees with the study of Aguilar-Marin et al. [47]. In addition, Tian et al. [17] reported an increase in propionic acid production and a decline in the relative abundance of specific methanogenic genera in the rumen of goat fed different levels of organic Se. These findings explain the decline of methanogens community and genus *Methanosphaera* in the rumen of supplemented sheep in our study.

Genus *Methanosphaera* is one of the major methane-producing genera in the gut of ruminant animals and it was declined in the rumen of Barki sheep when propionic acid was increased [16], which supports the previous results. Based on the findings of previous studies and the higher relative abundance of *Prevotella* and *Rikenellaceae RC9 gut group* in the rumen of supplemented groups, it could be speculated that Se supplementation could improve lignocellulose digestion and modulate the rumen methanogenic community.

The decline in the relative abundance of phylum Firmicutes might indicate that Se supplementation is not suitable for this phylum [18]. This phylum was dominated by *Oscillospiraceae NK4A214 group* and *Christensenellaceae R-7 group*. *Oscillospiraceae NK4A214 group* is uncultured bacteria that was detected in the rumen of reindeer and the role of this bacteria in the rumen is still unknown [48]. *Christensenellaceae R-7* has a potential role in fiber digestion and was linked positively with animal performance [44–49], however, the relative abundance of this group did not change due to Se supplementation. Phylum Synergistota has a potential role in Se utilization, which could explain its presence in sheep group SS and SeNSM [18].

It could be observed that SeNSM affected the relative abundance of some bacterial and archaeal groups compared with SS. For instance, the genus Oscillospiraceae NK4A214 group (phylum Firmicutes) and Rikenellaceae RC9 gut group (Phylum Bacteroidia) were higher in SeNSM compared to SS. Furthermore, the archaeal genus Methanosphaera was not detected in SeNSM group. These findings indicated that selenium sources affect the relative abundance of some rumen bacteria and archaea. Mainville et al. [23] explained that rumen microbial groups respond to organic or inorganic Se differently and the uptake of organic Se by rumen flora was higher than inorganic Se, which supports the current findings. Han et al. [50] explained that nano-Se contains more active centers than sodium selenite, which improves the biological activity and lowers the toxicity. Hendawy et al. [14] reported that inorganic Se has a shorter half-life compared to organic Se; therefore organic selenium can bind to bacterial protein and be stored in the body for later use.

## Conclusion

This study provides deep insight into the effect of Se source on the rumen microbial community of lactating Barki sheep. Selenium supplementation declined rumen methanogens and improved the abundance of some microbial groups that have a role in fiber digestion, which indicates that Se supplementation could improve dietary fiber digestibility and alleviating methane emission from the animal. Organic nano-Se did not exhibit a significant effect on the rumen microbiota compared to sodium selenite. However, considering the safety factor, it might be recommended to use organic nano-Se in animal diet.

## Data Availability

Datasets generated and/or analyzed during this study are included in this article version, and if required any further information related to the data involved in the manuscript can be obtained from the corresponding author upon reasonable request. All the sequences were deposited to the sequence read archive (SRA) under the accession number: PRJNA956058.

## References

[1] Spears JW (1994). Minerals in Forages. Forage quality, evaluation, and utilization.

[2] Overton TR, Yasui T (2014). Practical applications of trace minerals for dairy cattle. J Anim Sci..

[3] Khalil MM, Soltan YA, Abou Khadiga G, Elmahdy A, Sallam SM, Zommara MA, Rabee AE, Khattab IM (2023). Comparison of dietary supplementation of sodium selenite and bio-nanostructured selenium on nutrient digestibility, blood metabolites, antioxidant status, milk production, and lamb performance of Barki ewes. Anim Feed Sci Technol..

[4] Lu J, Holmgren A (2009). Selenoproteins. J Biol Chem..

[5] Hugejiletu H, Bobe G, Vorachek WR, Gorman ME, Mosher WD, Pirelli GJ, Hall JA (2013). Selenium supplementation alters gene expression profiles associated with innate immunity in whole-blood neutrophils of sheep. Biol Trace Elem Res..

[6] Helmer C, Hannemann R, Humann-Ziehank E, Kleinschmidt S, Koelln M, Kamphues J, Ganter M (2021). A case of concurrent molybdenosis, secondary copper, cobalt and selenium deficiency in a small sheep herd in northern Germany. Animals..

[7] Shini S, Sultan A, Bryden WL (2015). Selenium biochemistry and bioavailability: implications for animal agriculture. Agriculture..

[8] Ahuja A, Parmar D (2017). Role of minerals in reproductive health of dairy cattle: a review. Int J Livest Res..

[9] Pavlata L, Mišurová L, Pechová A, Dvořák R (2012). Comparison of organic and inorganic forms of selenium in the mother and kid relationship in goats. Czech J Anim Sci..

[10] Huang Q, Wang S, Yang X, Han X, Liu Y, Khan NA, Tan Z (2023). Effects of organic and inorganic selenium on selenium bioavailability, growth performance, antioxidant status and meat quality of a local beef cattle in China. Front Vet Sci..

[11] Malyugina S, Skalickova S, Skladanka J, Slama P, Horky P (2021). Biogenic selenium nanoparticles in animal nutrition: a review. Agric..

[12] Salam AY, IS EL-S, Metwally AM, El Hewaty AY, Mahmoud TA, Zommara MA (2021). Effect of selenium administration on reproductive outcome and biochemical parameters to ewes and their lambs. J Anim Poult Prod..

[13] Milewski S, Sobiech P, Błażejak-Grabowska J, Wójcik R, Żarczyńska K, Miciński J, Ząbek K (2021). The efficacy of a long-acting injectable selenium preparation administered to pregnant ewes and lambs. Animals..

[14] Hendawy AO, Sugimura S, Sato K, Mansour MM, El-Aziz A, Ayman H, Samir H, Islam M, Bostami ABM, Mandour AS, Elfadadny A (2022). Effects of selenium supplementation on rumen microbiota, rumen fermentation, and apparent nutrient digestibility of ruminant animals: a review. Ferment..

[15] Rabee AE, Kewan KZ, Sabra EA, El Shaer HM, Lamara M (2021). Rumen bacterial community profile and fermentation in Barki sheep fed olive cake and date palm byproducts. Peer J..

[16] Rabee AE, Kewan KZ, El Shaer HM, Lamara M, Sabra EA (2022). Effect of olive and date palm by-products on rumen methanogenic community in Barki sheep. AIMS Microbiol..

[17] Tian X, Wang X, Li J, Luo Q, Ban C, Lu Q (2022). The effects of selenium on rumen fermentation parameters and microbial metagenome in goats. Ferment..

[18] Cui X, Wang Z, Tan Y, Chang S, Zheng H, Wang H, Yan T, Guru T, Hou F (2021). Selenium yeast dietary supplement affects rumen bacterial population dynamics and fermentation parameters of tibetan sheep (Ovis aries) in alpine meadow. Front Microbiol..

[19] Kišidayová S, Mihaliková K, Siroka P, Čobanová K, Váradyová Z (2014). Effects of inorganic and organic selenium on the fatty acid composition of rumen contents of sheep and the rumen bacteria and ciliated protozoa. Anim Feed Sci Technol..

[20] Pino F, Heinrichs AJ (2016). Effect of trace minerals and starch on digestibility and rumen fermentation in diets for dairy heifers. J Dairy Sci..

[21] Naziroglu M, Aksakal M, Cay M, Celik S (1997). Effects of vitamin E and selenium on some rumen parameters in lambs. Acta Vet Hung..

[22] Shi L, Xun W, Yue W, Zhang C, Ren Y, Liu Q, Wang Q, Shi L (2011). Effect of elemental nano-selenium on feed digestibility, rumen fermentation, and purine derivatives in sheep. Anim Feed Sci Technol..

[23] Mainville AM, Odongo NE, Bettger WJ, McBride BW, Osborne VR (2009). Selenium uptake by ruminal microorganisms from organic and inorganic sources in dairy cows. Can J Anim Sci..

[24] Abd El-Razik EM, Soaud AA, ElKilani R, Habashy NR (2013). Distribution of selenium in some Egyptian soils. Egypt J Soil Sci..

[25] Mousaie A. Dietary supranutritional supplementation of selenium-enriched yeast improves feed efficiency and blood antioxidant status of growing lambs reared under warm environmental condition. Trop Anim Health Prod. 2021;53:138. 10.1007/s11250-021-02588-4 33486618

[26] Prokisch J, Zommara MA, inventors; Aliment Kft, assignee. Process for producing elemental selenium nanospheres. United States patent US 8,003,071. 2011.

[27] Kar T, Misra AK (2019). Therapeutic properties of whey used as fermented drink. Rev De Microbiol..

[28] Prokisch J, Sz’eles ´ E, Kov’acs B, Dar’oczy L, Zommara M (2008). Formation of metal selenium nanospheres in bacteria: is it a possible detoxification mechanism?. Cereal Res Commun..

[29] NRC (2007). Nutrient requirements of small ruminants: sheep, goats, Cervids, and New World camelids.

[30] AOAC. Official methods of analysis, 20th. Arlington: Association of Official Agricultural Chemists; 2006.

[31] Van Soest PV, Robertson JB, Lewis BA (1991). Methods for dietary fiber, neutral detergent fiber, and nonstarch polysaccharides in relation to animal nutrition. J. Dairy Sci..

[32] Elmer P. The THGA graphite furnace: techniques and recommended conditions. Recommended conditions for cadmium. Perkin Elmer Publication B. 1992;3210.

[33] El-Essawy AM, Anele UY, Abdel-Wahed AM, Abdou AR, Khattab IM (2021). Effects of anise, clove and thyme essential oils supplementation on rumen fermentation, blood metabolites, milk yield and milk composition in lactating goats. Anim Feed Sci Technol..

[34] Warner ACI (1964). Production of volatile fatty acids in the rumen: methods of measurement. Nutr Abstr Rev..

[35] Callahan BJ, McMurdie PJ, Rosen MJ, Han AW, Johnson AJA, Holmes SP (2016). DADA2: high-resolution sample inference from Illumina amplicon data. Nat Methods..

[36] Ferro C, Florindo HF, Santos HA (2021). Selenium nanoparticles for biomedical applications: from development and characterization to therapeutics. Adv Healthc Mater..

[37] Xun W, Shi L, Yue W, Zhang C, Ren Y, Liu Q (2012). Effect of high-dose nano-selenium and selenium–yeast on feed digestibility, rumen fermentation, and purine derivatives in sheep. Biol Trace Elem Res..

[38] Wei JY, Wang J, Liu W, Zhang KZ, Sun P (2019). Effects of different selenium supplements on rumen fermentation and apparent nutrient and selenium digestibility of mid-lactation dairy cows. J Dairy Sci..

[39] Taheri Z, Karimi S, Mehrban H, Moharrery A. Supplementation of different selenium sources during early lactation of native goats and their effects on nutrient digestibility, nitrogen and energy status. J. Appl. Anim. Res. 2018;46:64-68.

[40] Kim J, Soest PJV, Combs GF (1997). Studies on the effects of selenium on rumen microbial fermentation in vitro. Biol Trace Elem Res..

[41] Naas AE, Mackenzie AK, Mravec J, Schückel J, Willats WGT, Eijsink VGH, Pope PB (2014). Do rumen Bacteroidetes utilize an alternative mechanism for cellulose degradation?. MBio..

[42] Russell JB, Rychlik JL (2001). Factors that alter rumen microbial ecology. Science..

[43] Xue MY, Sun HZ, Wu XH, Liu JX, Guan LL (2020). Multi-omics reveals that the rumen microbiome and its metabolome together with the host metabolome contribute to individualized dairy cow performance. Microbiome..

[44] Huang C, Ge F, Yao X, Guo X, Bao P, Ma X, Wu X, Chu M, Yan P, Liang C (2021). Microbiome and metabolomics reveal the effects of different feeding systems on the growth and ruminal development of yaks. Front Microbiol..

[45] Rabee AE, Forster R, Elekwachi C, Sabra E, Lamara M (2020). Comparative analysis of the metabolically active microbial communities in the rumen of dromedary camels under different feeding systems using total rRNA sequencing. PeerJ..

[46] Piao H, Lachman M, Malfatti S, Sczyrba A, Knierim B, Auer M, Tringe SG, Mackie RI, Yeoman CJ, Hess M (2014). Temporal dynamics of fibrolytic and methanogenic rumen microorganisms during in situ incubation of switchgrass determined by 16S rRNA gene profiling. Front Microbiol..

[47] Aguilar-Marin SB, Betancur-Murillo CL, Isaza GA, Mesa H, Jovel J (2020). Lower methane emissions were associated with higher abundance of ruminal Prevotella in a cohort of Colombian buffalos. Bmc Microbial..

[48] Yildirim E, Ilina L, Laptev G, Filippova V, Brazhnik E, Dunyashev T, Dubrovin A, Novikova N, Tiurina D, Tarlavin N, Laishev K (2021). The structure and functional profile of ruminal microbiota in young and adult reindeers (Rangifer tarandus) consuming natural winter-spring and summer-autumn seasonal diets. PeerJ..

[49] Bach A, López-García A, González-Recio O, Elcoso G, Fàbregas F, Chaucheyras-Durand F, Castex M (2019). Changes in the rumen and colon microbiota and effects of live yeast dietary supplementation during the transition from the dry period to lactation of dairy cows. J Dairy Sci..

[50] Han L, Pang K, Fu T, Phillips CJ, Gao T (2021). Nano-selenium supplementation increases selenoprotein (Sel) gene expression profiles and milk selenium concentration in lactating dairy cows. Biol Trace Elem Res..

